# Urinary ATP and visualization of intracellular bacteria: a superior diagnostic marker for recurrent UTI in renal transplant recipients?

**DOI:** 10.1186/2193-1801-3-200

**Published:** 2014-04-23

**Authors:** Stephen P Kelley, Holly R Courtneidge, Rebecca E Birch, Alberto Contreras-Sanz, Mark C Kelly, Jerome Durodie, Claire M Peppiatt-Wildman, Christopher K Farmer, Michael P Delaney, James Malone-Lee, Mark A Harber, Scott S Wildman

**Affiliations:** Medway School of Pharmacy, The Universities of Kent and Greenwich at Medway, Central Avenue, Chatham Maritime, Kent ME4 4TB UK; Centre for Nephrology, UCL Medical School, Royal Free Campus, London, UK; Renal Unit, East Kent Hospitals University NHS Foundation Trust, Canterbury, Kent UK; Research Department of Clinical Physiology, Whittington Campus, University College London Medical School, London, UK

**Keywords:** Intracellular bacteria, IBC, Pyuria, Urinary ATP, Bladder, Acridine orange stain

## Abstract

Renal transplant recipients (RTR) are highly susceptible to urinary tract infections (UTIs) with over 50% of patients having at least one UTI within the first year. Yet it is generally acknowledged that there is considerable insensitivity and inaccuracy in routine urinalysis when screening for UTIs. Thus a large number of transplant patients with genuine urine infections may go undiagnosed and develop chronic recalcitrant infections, which can be associated with graft loss and morbidity. Given a recent study demonstrating ATP is released by urothelial cells in response to bacteria exposure, possibly acting at metabotropic P2Y receptors mediating a proinflammatory response, we have investigated alternative, and possibly more appropriate, urinalysis techniques in a cohort of RTRs.

Mid-stream urine (MSU) samples were collected from 53 outpatient RTRs. Conventional leukocyte esterase and nitrite dipstick tests, and microscopic pyuria counts (in 1 μl), ATP concentration measurements, and identification of intracellular bacteria in shed urothelial cells, were performed on fresh unspun samples and compared to ‘gold-standard’ bacterial culture results.

Of the 53 RTRs, 22% were deemed to have a UTI by ‘gold-standard’ conventional bacteria culture, whereas 87%, 8% and 4% showed evidence of UTIs according to leukocyte esterase dipstick, nitrite dipstick, and a combination of both dipsticks, respectively. Intracellular bacteria were visualized in shed urothelial cells of 44% of RTRs, however only 1 of the 23 RTRs (44%) was deemed to have a UTI by conventional bacteria culture. A significant association of the ‘gold-standard’ test with urinary ATP concentration combined with visualization of intracellular bacteria in shed urothelial cells was determined using the Fisher’s exact test.

It is apparent that standard bedside tests for UTIs give variable results and that seemingly quiescent bacteria in urothelial cells are very common in RTRs and may represent a focus of subclinical infection. Furthermore, our results suggest urinary ATP concentration combined with detection of intracellular bacteria in shed urinary epithelial cells may be a sensitive means by which to detect ‘occult’ infection in RTRs.

## Background

Renal transplant recipients (RTRs) are susceptible to urinary tract infections (UTIs), which are the commonest post transplant infections, and approximately 40% of patients go on to get recurrent UTIs (Mitra and Alangaden 2011). In a review of 30,000 patients, those with post-transplant UTIs had a 3-fold greater risk of death and a 2-fold greater risk of graft loss than those without (Abbott et al. 2004).

The standard routine clinical-practice tests currently used to diagnose UTIs regularly misdiagnose infection and antibiotics are often not prescribed when appropriate (Franz and Horl 1999), which may explain the recurrence in many cases (Manges et al. 2001). Approximately 65% of recurring UTIs seem to be caused by the same microorganism, and evidence suggests this may be due to chronic sub-clinical ‘intracellular’ bladder wall infection (Anderson et al. 2003). This evidence is supported by data demonstrating that conventional diagnostic tests only give a positive result in 14% of all patients who were subsequently diagnosed with recurrent UTIs, highlighting the need for improvement (van Haarst et al. 2001;Arinzon et al. 2009).

Failure to diagnose a common UTI, such as cystitis, may increase UTI severity by allowing progression from the lower to the upper urinary tract. One study found that up to 33% of RTRs with UTIs went on to develop acute pyelonephritis (APN) (Valera et al. 2006). Without treatment, APN can lead to bacteraemia, renal failure and sepsis (Rubin 1993). Acute kidney infection is also an independent risk factor for the deterioration of graft function and may increase the risk of subsequent acute rejection (Pelle et al. 2007). Given its severity and possible implications on graft function in RTRs, it is clear to see why early detection to facilitate effective treatment of UTIs is at the crux of the issue.

Unfortunately, UTI diagnosis is often problematical due to the absence of symptoms. This is an issue especially relevant to RTRs, who are more likely to suffer from clinically asymptomatic UTIs than their non-immunocompromised counterparts (Saemann and Horl 2008). As yet, this is an unavoidable consequence of the immunosuppressive drugs taken post-transplantation, which prevent the mounting of a conventional inflammatory response to infection (Gangappa et al. 2008). In addition to immunosuppressive drugs, RTRs routinely receive combination antibiotic therapy (e.g. trimethoprim and sulfamethoxazole [Trim/Sul]) for the first 3–12 months (in the UK) following transplantation to prevent *Pneumocystis jirovecia* infection. However, it is believed that as a result of antibiotic resistance, and/or the low doses routinely used, Trim/Sul is not completely effective against UTIs, and this may result in persistent sub-clinical infections. Ultimately there is an inherent difficulty in identifying UTIs in RTRs.

UTI is responsible for approximately 40-50% of all infectious complications post transplantation and is also found to be a more common affliction in RTRs than in the general population (Chan et al. 1990;Rabkin et al. 1998;Glazier et al. 1998). For these reasons, an effective diagnosis would present significant benefits. Previous studies have suggested alternative urinalysis for UTI detection (e.g. quantification of microscopic pyuria, visualization of intracellular bacteria in shed urothelial cells, urinary interleukin [IL] levels, and urinary ATP concentration) and although reported to be less powerful that the current ‘gold standard’ in the general population, the possibility exists that they may be appropriate for an immunocompromised RTR cohort (Stamm 1983;Lundin et al. 1989;Miliotis 1991;Ivancic et al. 2008). In support of this notion, Säve and Persson have recently demonstrated that ATP is released by urothelial cells in response to uropathogenic *Escherichia coli* exposure, possibly acting at metabotropic P2Y receptors mediating a proinflammatory IL-8 response (Save and Persson 2010). That the IL-8 response is likely dampened in immunosuppressed RTRs does not, to our minds, infer that ATP release from infected urothelial cells is also suppressed.

By investigating alternative diagnostic methods (i.e. quantification of microscopic pyuria and urinary ATP concentration, and visualization of intracellular bacteria in shed urothelial cells; see *Methods*) in this cross-sectional, one-time sampling, preliminary investigation we hope to shed light on a key issue involving both patient welfare and economic impact. We hypothesize, bacterial colonization of shed urothelial cells and high levels of urinary ATP (i.e. >50 nmol/l; presumably as a result of a proinflammatory response involving the purinergic system) is a powerful marker of UTI in RTRs when compared to the current ‘gold standard’ culture test.

## Methods

53 patients who underwent renal transplantation at the Royal Free Hospital, London, UK, between the years 2009–2012 were included in this study. Transplant recipients were recruited within 2–4 weeks following transplantation (i.e. >2 weeks before stent removal). All subjects were monitored for UTIs upon recruitment and urinalysis was performed on mid-stream urine (MSU) samples.

Urinalysis comprised of *i*) routine clinical-practice bedside leukocyte esterase and nitrite dipstick tests (read using a bedside automated analyzer), *ii*) routine clinical-practice bacterial culture on Columbia blood agar plates, *iii*) non-routine quantification of microscopic pyuria in 1 μl of fresh unspun urine using a haemocytometer as previously described (Stamm 1983), *iv*) non-routine quantification of urinary ATP concentration in 50 μl of fresh unspun urine, using a luciferin/luciferase assay (ATP Bioluminiscence Assay Kit, detection range 2x10^−10^ – 2x10^−4^ M ATP; Sigma, Poole, UK) and a luminometer (Synergy 2, Biotek, Winooski, USA), as previously described (Lundin et al. 1989), and *v*) non-routine identification of bacteria present both in the intracellular domain, and on the surface, of shed transitional epithelial (urothelial) cells in an unfixed cytospin cell preparation (800 rpm, 5 min, at room temperature, prepared from 100 μl of fresh urine; Sandon Cytospin 4, York, UK), using acridine orange and crystal violet stains (Miliotis 1991) and fluorescence microscopy (Leica Nicrosystems GmgH, DMIRB, Wetlar, Germany). Immunocytochemistry and fluorescence microscopy was retrospectively performed on samples with confirmed intracellular bacteria using anti-uroplakin III (UPIII, 1:200, overnight incubation; Santa Cruz Biotechnology, Santa Cruz, USA) and a FITC conjugated secondary antibody (1:1000 for 2 h; 1:1000; Invitrogen, Paisley, UK) to verify the cells as urothelial cells (as opposed to other epithelial cells e.g. of renal or vaginal origin).

A two-tailed Fisher’s exact test was used to test for associations between routine clinical-practice ‘gold-standard’ culture tests and each of the following tests: leukocyte esterase dipstick, nitrite dipstick, microscopic pyuria, urinary ATP concentration, and visualization of intracellular bacteria in shed urothelial cells. The performance of the various diagnostic tests were evaluated by the following metrics: sensitivity, specificity (i.e., positive predictive value [PPV], positive likelihood ratio [LR+], negative likelihood ratio [LR-], accuracy, Youden's index and the diagnostic odds ratio [DOR]). The formulae used for each metric are summarized in Table 1. The metrics and associated confidence intervals were calculated using Microsoft Excel and Instat (GraphPad Software Inc, La Jolla, USA).

**Table 1:** **Formulae for test metrics used to evaluate the performance of diagnostic tests**

Test Metric	Formula
Sensitivity	TP/(TP + FN)
Specificity	TN/(TN + FP)
Positive Predictive Value (PPV)	TP/(TP + FP)
Positive Likelihood Ratio (LR+)	Sensitivity/(1-Specificity)
Negative Likelihood Ratio (LR-)	(1-Sensitivity)/Specificity
Accuracy	(TP + TN)/(TP + TN + FP + FN)
Youden’s Index	Sensitivity + Specificity −1
Diagnostic Odds Ratio (DOR)	(TP/FN)/(FP/TN)

Key: TP, TN, FP, and FN denote the number of true positives, true negatives, false positives, and false negatives, respectively.

An ethics board, specifically the Moorfields and Whittington Hospitals Research Ethics Committee, approved this study.

## Results and discussion

A total of 53 urine samples were collected from 53 renal transplant recipients. The majority of patients (n = 41; 77%) tested negative for the presence of bacteria above the threshold of 10^5^ colony-forming units per ml (CFU ml^−1^; deemed the ‘gold-standard’ for diagnosing a UTI). Interestingly, 50% of those testing positive (n = 6) were asymptomatic – further highlighting the high incidence of clinically asymptomatic UTIs in immunocompromised RTRs.

Almost the entire patient group (n = 46; 87%) tested positive for leukocyte esterase (i.e. gave a reading of +2 or +3 according to the bedside automated analyzer). However of those testing positive, only 13% (n = 6) also tested positive for the presence of bacteria above 10^5^ CFU ml^−1^. A significant association of the ‘gold-standard’ test and leukocyte esterase test was determined using the Fisher’s exact test (P < 0.01). In contrast to leukocyte esterase, a minority of patients (n = 4; 8%) tested positive for nitrites. Of those testing positive for nitrites, 50% (n = 2) also tested positive for the presence of bacteria above 10^5^ CFU ml^−1^. The majority (80%, n = 39) of those testing negative for nitrites were also culture negative. When combining leukocyte esterase and nitrite dipstick tests, only 7 patients (14%) showed parity (i.e. both tests were positive, or both tests were negative). Zero patients tested positive for both dipsticks and positive for the presence of bacteria above 10^5^ CFU ml^−1^, and only 2% (n = 1) tested negative for both dipsticks and bacteria levels below 10^5^ CFU ml^−1^.

The majority of patients (n = 44; 83%) were found to have microscopic pyuria levels of ≥10 white blood cells (WBC) in 1 μl of fresh unspun urine; proposed as indicative of a UTI (Stamm 1983). Of those found to have microscopic pyuria, 82% (n = 36) had pyuria levels ranging from 10–40 WBC in 1 μl of fresh unspun urine.

A concentration of ATP, ≥50 nM, in 50 μl of fresh unspun urine (i.e. ≥50 nmol/l) is proposed as indicative of a UTI (Lundin et al. 1989). A minority of patients (n = 3; 6%) was found to have urinary ATP levels ≥50 nmol/l. Of those testing positive, all 3 also tested positive for the presence of bacteria above 10^5^ CFU ml^−1^. Conversely, 77% patients (n = 41) with a urinary concentration <50 nmol/l tested negative for the presence of bacteria (i.e. <10^5^ CFU ml^−1^). A significant association of the ‘gold-standard’ test and urinary ATP concentration was determined using the Fisher’s exact test (P < 0.01).

**Figure 1:**
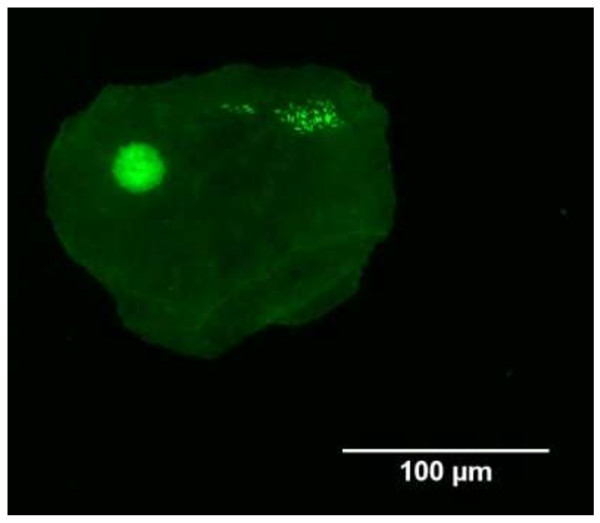
**Shed urothelial cell with evidence of intracellular bacteria.** Intracellular localization of bacteria was confirmed by staining with acridine orange and counterstaining with crystal violet; viewed using the x60 objective on a fluorescence microscope. Immunocytochemistry with anti-UPIII (FITC; green) confirmed that cells were urothelial.

Cytospin urine samples from the majority of patients (n = 41; 77%) contained >3 urothelial cells. Of those found to have shed urothelial cells, the majority (n = 23; 56%) had urothelial cells that contained intracellular bacteria (determined using acridine orange (Miliotis 1991); see Figure 1). Interestingly of the 23 patients with urinary epithelial cells containing bacteria, 22 went on to be classified as bacteria culture negative (i.e. <10^5^ CFU ml^−1^) and consequently deemed not to have a UTI. It was qualitatively noted that if intracellular bacteria were identified in one urothelial cell it would also be identified in accompanying shed urothelial cells. A significant association of the ‘gold-standard’ test and the presence of intracellular bacteria in shed urothelial cells were determined using the Fisher’s exact test (P < 0.01). When combining the concentration of ATP, ≥50 nM (in 50 μl of fresh unspun urine) with the observation of urinary epithelial cells containing bacteria (in 100 μl of fresh urine), one patient (2%) showed parity. In contrast, a concentration of ATP <50 nM and an absence of intracellular bacteria in shed urinary epithelial cells was observed in 17 patients (32%). Of the 17 patients, all were culture negative (<10^5^ CFU ml^−1^). A significant association of the ‘gold-standard’ test and combined urinary ATP concentration and evidence of intracellular bacteria in shed epithelial cells was determined using the Fisher’s exact test (P < 0.01). The main results of this study are summarized in Table 2.

**Table 2:** **Contingency tables detailing comparison of surrogate markers to the ‘gold-standard’ for UTI diagnosis (bacterial culture), in a cohort of renal transplant recipients**

(1) Leukocyte esterase*			(2) Nitrite			(3) Combined dipsticks		
	Culture positive	Culture negative		Culture positive	Culture negative		Culture positive	Culture negative
**Leukocyte positive**	6 (11%)	40 (76%)	**Nitrite positive**	2 (4%)	2 (4%)	**Both positive**	0 (4%)	2 (4%)
**Leukocyte negative**	6 (11%)	1 (2%)	**Nitrite negative**	10 (19%)	39 (73%)	**Both negative**	4 (8%)	1 (2%)
**(4) Pyuria**			(5) ATP*			(6) IB*		
	**Culture positive**	**Culture negative**		**Culture positive**	**Culture negative**		**Culture positive**	**Culture negative**
**Pyuria positive**	9 (17%)	35 (66%)	**ATP >50 nmol/l**	3 (6%)	0 (0%)	**IB positive**	1 (2%)	22 (41%)
**Pyuria negative**	3 (6%)	6 (11%)	**ATP <50 nmol/l**	9 (17%)	41 (77%)	**IB negative**	11 (21%)	19 (36%)
(7) Combined ATP and IB**								
	**Culture positive**	**Culture negative**						
**ATP >50, IB positive**	1 (2%)	0 (0%)						
**ATP <50, IB negative**	0 (0%)	18 (34%)						

*denotes Fisher’s exact test <0.05. **denotes Fisher’s exact test =0.0556 (not quite significant). See Methods for an explanation of the surrogate markers used. Key: IB, intracellular bacteria.

Data not included in the contingency tables: with respect to combined dipstick tests; 6 patients (10%) were leukocyte positive/nitrite negative and culture positive; 37 patients (70%) were leukocyte positive/nitrite negative and culture negative; 2 patients (4%) where leukocyte negative/nitrite positive and culture positive; 1 patient (2%) was where leukocyte negative/nitrite positive and culture negative. With respect to combined ATP and IB; 9 patients (17%) were IB positive/ATP <50 and culture positive; 22 patients (41%) were IB positive/ATP <50 and culture negative; 3 patients (6%) were IB negative/ATP >50 and culture positive; 0 patients were IB negative/ATP >50 and culture negative.

Various urinalysis techniques were evaluated across a range of quantitative indicators. Measurement of urinary ATP concentration, when compared to the ‘gold-standard’ , evidenced strong effectiveness as a diagnostic test for UTI in renal transplant patients. This was reflected in the high degree of specificity, PPV, LR+, LR-, and accuracy of the diagnostic test. Additionally, the urinary ATP concentration diagnostic test evidenced strong effectiveness in both the Youden’s index and DOR, both important measures of diagnostic test accuracy and performance (Glas et al. 2003). ATP concentration outperformed all the other diagnostic tests on these metrics with the sole exception of urinary ATP concentration combined with identification of intracellular bacteria. This combined diagnostic screen outperformed all the diagnostic tests for UTI in this study. This was evidenced in the unison values obtained for selectivity, specificity, PPV, and accuracy, in addition to the comparatively strong measurements of 1 and 105 obtained for the Youden’s index and DOR, respectively. Statistical analyses of the main results in this study are summarized in Table 3.

**Table 3:** **Comparison of variables to determine the power of surrogate markers to the ‘gold-standard’ for UTI diagnosis (bacterial culture), in a cohort of renal transplant recipients**

	Sensitivity (95% CL)	Specificity (95% CL)	PPV (95% CL)	LR+	LR-	Accuracy	Youden’s index	DOR (95% CL)
**(1) Leukocyte esterase**	0.500 (0.21-0.79)	0.024 (0.00-0.13)	0.130 (0.05-0.26)	0.513	20.5	0.132	−0.476	0.025 (0.00-0.25)
**(2) Nitrite**	0.167 (0.02-0.48)	0.951 (0.83-0.99)	0.500 (0.07-0.93)	3.417	0.876	0.774	0.118	3.900 (0.49-31.22)
**(3) Combined dipsticks**	0.000 (0.00-0.60)	0.333 (0.01-0.91)	0.000 (0.00-0.84)	0.000	3.000	0.143	−0.667	0.067 (0.00-2.33)
**(4) Pyuria**	0.750 (0.43-0.95)	0.146 (0.06-0.29)	0.205 (0.10-0.35)	0.879	1.708	0.283	−0.104	0.514 (0.11-2.47)
**(5) ATP**	0.250 (0.05-0.57)	1.000 (0.91-1.00)	1.000 (0.29-1.00)	Infinity	0.750	0.830	0.250	30.580 (1.45-643.50)
**(6) IB**	0.083 (0.00-0.38)	0.463 (0.31-0.63)	0.043 (0.00-0.22)	0.155	1.978	0.377	−0.453	0.079 (0.01-0.67)
**(7) Combined ATP and IB**	1.000 (0.03-1.00)	1.000 (0.81-1.00)	1.000 (0.03-1.00)	Infinity	0.000	1.000	1.000	105.000 (1.48-7441.80)

Key: PPV, positive predictive value; LR+, positive likelihood ratio; LR-, negative likelihood ratio; DOR, diagnostic odds ratio; IB, intracellular bacteria.

Putting aside RTRs, it is apparent that the standard clinical methods for diagnosing UTIs are fallible and this may result in delayed or missed diagnosis with significant clinical consequences. There remains a high range of variability between methods, the ‘gold-standard’ of bacterial culturing is a fundamentally flawed process and the sensitivity and specificity of dipstick tests (alone or combined) leave much to be desired. Here we highlight this as especially relevant to RTRs.

### The inadequacy of current UTI diagnosis in RTRs

Standard laboratory methods for analysing MSU samples are set at a threshold of 10^5^ CFU ml^−1^ (Stamm et al. 1982). Hooton & Stamm demonstrated that laboratory MSU cultures fail to detect over 50% of genuine infections in women when using this threshold (Hooton and Stamm 1997). In part this may be explained by current culture methods assuming dominant pathogenicity from the *Enterobacteriaceae* species, notably *Escherichia coli*, and owing to this presumption, MSU culture is performed on selective chromogenic medium for *Enterobacteriaceae* under aerobic conditions. As such, anaerobic bacteria present will not be cultivated and some species of aerobic bacteria may be overlooked. The same is inherently true for RTRs.

Numerous studies have described the rapid dipstick tests as unreliable. Although the nitrite test is reported as having excellent specificity (0.85-0.98), its sensitivity (0.45-0.60) is questionable (Deville et al. 2004). When compared to bacteria culture, previous studies have demonstrated a failure to detect between 20-60% of UTIs (Deville et al. 2004;Semeniuk and Church 1999). Here, using a cohort of RTRs, we report similar specificity values (0.83-0.99), however, dissimilar sensitivity (0.02-0.48). Interestingly, none of the RTRs presenting with intracellular bacteria in this study gave a positive nitrite result (data not shown). Conversely, the leukocyte esterase dipstick has greater sensitivity (0.21-0.79), but the specificity (0.00-0.13) seems poor for RTRs. The values for sensitivity compare favourably to values reported for non-RTR cohorts (0.48-0.86), however as with the nitrite test, specificity differs (0.17-0.93 (Deville et al. 2004)). Perhaps not surprisingly, in our results 87% of RTRs had a UTI according to the leukocyte esterase dipstick test. It is noteworthy that a positive leukocyte esterase dipstick test may indicate cystitis (of a non-bacterial origin) and interstitial nephritis. Despite the recommendation that nitrite and leukocyte esterase dipsticks should be used in combination, this may not always be the case. Furthermore, with respect to the RTRs, this does little to improve the diagnostic power of these tests (see Table 3).

Our data confirms the notion that many of the described inadequacies in current urinalysis become more apparent when studying RTRs. The consequences of misdiagnosis in this cohort are far reaching, with APN being diagnosed in approximately a third of all patients with a UTI at one time or another post-transplant (Valera et al. 2006).

### Promising new tests for UTI diagnosis in RTRs

Of great interest are our results for urinary ATP concentration as a marker of UTIs in RTRs, which showed a significant association to ‘gold-standard’ bacterial culture results. Similar to the principle behind the nitrite dipstick, these data suggests that urinary ATP <50 nmol/l may be a useful tool in *ruling out* a UTI (see Table 3). The concentration of ATP from those RTRs that were subsequently found to be culture positive was ~10-fold lower than those seen in the previous study by Lundin *et al.* using a cohort of non-transplant patients (Lundin et al. 1989). We speculate the cause of a lower urinary ATP concentration is due to immunosuppression and fewer WBCs in the urine, as bacterial infection also induces the release of ATP from immune cells (Rizzo et al. 2009). Although not investigated here, there may be merit in re-evaluating the concentration of ATP (currently set at ≥50 nM, in 50 μl of fresh unspun urine, by Lunden *et al.* (Lundin et al. 1989)) that is indicative of a UTI with special reference to RTRs. Although haematuria was not evident in these patients, it is noteworthy that blood ATP concentration, in healthy subjects at least, can be relatively high 200 nM - 600 μM (Chida et al. 2013;Praetorius and Leipziger 2009). Since the major source of ATP in blood is red blood cell, haematuria may exclude urinary ATP concentration as a marker of UTIs in RTRs.

It has been proposed that many recalcitrant, and possible recurrent, UTIs are the result of an underlying infection caused by quiescent intracellular bacteria present in the transitional cell layer of the urothelium (Anderson et al. 2003), this may be most apposite for immunosuppressed RTRs. When comparing the proportion of RTRs with intracellular bacteria, with a similar study using patients with no other complications aside from UTIs we see a marked difference in results. We find 44% of our RTR cohort to have intracellular bacteria, whereas Rosen *et al.* found intracellular bacteria in just 18% of their UTI cohort (Rosen et al. 2007). This suggests a greater prevalence of intracellular bacteria in RTRs, and may account for the higher incidence of recurrent UTIs in RTR. This could be explained in part by these patients being immunosuppressed and having a reduced defense against invading bacteria. The primary defence against uropathogenic bacteria are phagocytic neutrophils recruited from the bloodstream directly to the site of invading bacteria (Kobayashi et al. 2003). Interestingly, we see that the urinary WBC count is significantly decreased in RTRs when compared to the ‘normal’ UTI patients (data not shown).

Perhaps the most important finding of the current study is the superior diagnostic ability of combining urinary ATP concentration with evidence of intracellular bacteria in shed urothelial cells compared to the current gold-standard (albeit an inadequate gold-standard). In our cohort of RTRs this combination yielded encouraging sensitivity and specificity values, as well as unrivalled PPV, LR+, accuracy, Youden’s index and DOR (see Table 2). These results suggest a low urinary ATP concentration and an absence of intracellular bacteria in shed urinary epithelial cells may be a powerful tool for ruling out UTIs (symptomatic, asymptomatic, or sub-clinical) and/or the likelihood of recurrent UTIs in RTRs (i.e. a negative diagnostic of UTI). ‘Ruling out’ being analogous to current cytomegalovirus (CMV) tests (using polymerase chain reaction [PCR] technology) in transplantation. However, in the current investigation we hypothesised: bacterial colonization of shed urothelial cells and high levels of urinary ATP (>50 nmol/l; presumably as a result of a proinflammatory response involving the purinergic system) is a powerful marker of UTI in RTRs when compared to the current ‘gold standard’ culture test. We believe that a subsequent larger longitudinal study into the natural history of UTIs in RTRs will further substantiate/prove our hypothesis. Furthermore, it will be interesting to see if this combined diagnostic methodology (ATP and intracellular bacteria), if introduced, would predict patients who subsequently go on to develop clinically important UTIs.

## Conclusions

In conclusion, we provide evidence of urinary tract disease in RTRs when routine clinical tests are negative, and thereby validate the suspicion of missed diagnosis. We propose that concealed infection through intracellular bacterial colonization of urothelial cells may account for recurrent UTIs seen in RTRs, which presents a real and serious concern. Finally we propose additional/alternative urinalysis for diagnosing UTIs in RTRs. We now need to build on this cross-sectional, one-time sampling, preliminary investigation by following RTRs for a sustained period, also investigating reproducibility (i.e. how often you can repeat the test in the same patients with the same results), and perhaps investigate the outcomes of those RTRs following treatment regimes based on standard UTI detection practice and the novel techniques we propose (i.e. urinary ATP concentration and evidence of intracellular bacteria in shed urothelial cells).

## Authors’ information

CKF, MPD and MAH are consultant nephrologists. JD is a pharmacist, microbiologist and former clinical microbiologist. SSW and CMP-W are physiologists specialising in the urinary system. SPK is a pharmacologist and statistician. JM-L is a clinical Professor of Medicine specializing in urinary incontinence. HRC, REB, AC-S, and MCK are/were PhD students.

SPK and HRC are joint first author.

## References

[CR1] Abbott KC, Swanson SJ, Richter ER, Bohen EM, Agodoa LY, Peters TG, Barbour G, Lipnick R, Cruess DF (2004). Late urinary tract infection after renal transplantation in the United States. Am J Kidney Dis.

[CR2] Anderson GG, Palermo JJ, Schilling JD, Roth R, Heuser J, Hultgren SJ (2003). Intracellular bacterial biofilm-like pods in urinary tract infections. Science.

[CR3] Arinzon Z, Peisakh H, Shuval I, Shabat S, Berner YN (2009). Detection of urinary tract infection (UTI) in long-term care setting: is the multireagent strip an adequate diagnostic tool?. Arch Gerontol Geriatr.

[CR4] Chan PC, Cheng IK, Wong KK, Li MK, Chan MK (1990). Urinary tract infections in post-renal transplant patients. Int Urol Nephrol.

[CR5] Chida J, Ono R, Yamane K, Hiyoshi M, Nishimura M, Onodera M, Nakataki E, Shichijo K, Matushita M, Kido H (2013). Blood lactate/ATP ratio, as an alarm index and real-time biomarker in critical illness. PLoS One.

[CR6] Deville WL, Yzermans JC, van Duijn NP, Bezemer PD, van der Windt DA, Bouter LM (2004). The urine dipstick test useful to rule out infections A meta-analysis of the accuracy. BMC Urol.

[CR7] Franz M, Horl WH (1999). Common errors in diagnosis and management of urinary tract infection. I. Pathophysiology and diagnostic techniques. Nephrol Dial Transplant.

[CR8] Gangappa S, Kokko KE, Carlson LM, Gourley T, Newell KA, Pearson TC, Ahmed R, Larsen CP (2008). Immune responsiveness and protective immunity after transplantation. Transpl Int.

[CR9] Glas AS, Lijmer JG, Prins MH, Bonsel GJ, Bossuyt PMM (2003). The diagnostic odds ratio: a single indicator of test performance. J Clin Epidemiol.

[CR10] Glazier DB, Jacobs MG, Lyman NW, Whang MI, Manor E, Mulgaonkar SP (1998). Urinary tract infection associated with ureteral stents in renal transplantation. Can J Urol.

[CR11] Hooton TM, Stamm WE (1997). Diagnosis and treatment of uncomplicated urinary tract infection. Infect Dis Clin North Am.

[CR12] Ivancic V, Mastali M, Percy N, Gornbein J, Babbitt JT, Landaw EM, Bruckner DA, Churchill BM, Haake DA (2008). Rapid antimicrobial susceptibility determination of uropathogens in clinical urine specimens by use of ATP bioluminescence. J Clin Microbiol.

[CR13] Kobayashi SD, Voyich JM, DeLeo FR (2003). Regulation of the neutrophil-mediated inflammatory response to infection. Microbes Infect.

[CR14] Lundin A, Hallander H, Kallner A, Lundin UK, Osterberg E (1989). Bacteriuria testing by the ATP method as an integral part in the diagnosis and therapy of urinary tract infection (UTI). J Biolumin Chemilumin.

[CR15] Manges AR, Johnson JR, Foxman B, O’Bryan TT, Fullerton KE, Riley LW (2001). Widespread distribution of urinary tract infections caused by a multidrug-resistant Escherichia coli clonal group. N Engl J Med.

[CR16] Miliotis MD (1991). Acridine orange stain for determining intracellular enteropathogens in HeLa cells. J Clin Microbiol.

[CR17] Mitra S, Alangaden GJ (2011). Recurrent urinary tract infections in kidney transplant recipients. Curr Infect Dis ReP.

[CR18] Pelle G, Vimont S, Levy PP, Hertig A, Ouali N, Chassin C, Arlet G, Rondeau E, Vandewalle A (2007). Acute pyelonephritis represents a risk factor impairing long-term kidney graft function. Am J Transplant.

[CR19] Praetorius HA, Leipziger J (2009). ATP release from non-excitable cells. Purinergic Signal.

[CR20] Rabkin DG, Stifelman MD, Birkhoff J, Richardson KA, Cohen D, Nowygrod R, Benvenisty AI, Hardy MA (1998). Early catheter removal decreases incidence of urinary tract infections in renal transplant recipients. Transplant Proc.

[CR21] Rizzo R, Ferrari D, Melchiorri L, Stignani M, Gulinelli S, Bacordi OR, Di Virgilio F (2009). Extracellular ATP acting at the P2X7 receptor inhibits secretion of soluble HLA-G from human monocytes. J Immunol.

[CR22] Rosen DA, Hooton TM, Stamm WE, Humphrey PA, Hultgren SJ (2007). Detection of intracellular bacterial communities in human urinary tract infection. Plos Med.

[CR23] Rubin RH (1993). Infectious disease complications of renal transplantation. Kidney Int.

[CR24] Saemann M, Horl WH (2008). Urinary tract infection in renal transplant recipients. Eur J Clin Invest.

[CR25] Save S, Persson K (2010). Extracellular ATP and P2Y receptor activation induce a proinflammatory host response in the human urinary tract. Infect Immun.

[CR26] Semeniuk H, Church D (1999). Evaluation of the leukocyte esterase and nitrite urine dipstick screening tests for detection of bacteriuria in women with suspected uncomplicated urinary tract infections. J Clin Microbiol.

[CR27] Stamm WE (1983). Measurement of pyuria and its relation to bacteriuria. Am J Med.

[CR28] Stamm WE, Counts GW, Running KR, Fihn S, Turck M, Holmes KK (1982). Diagnosis of Coliform Infection in Acutely Dysuric Women. N Engl J Med.

[CR29] Valera B, Gentil MA, Cabello V, Fijo J, Cordero E, Cisneros JM (2006). Epidemiology of urinary infections in renal transplant recipients. Transplant Proc.

[CR30] van Haarst EP, van Andel G, Heldeweg EA, Schlatmann TJ, van der Horst HJ (2001). Evaluation of the diagnostic workup in young women referred for recurrent lower urinary tract infections. Urology.

